# Real-World Effectiveness of Seasonal Influenza Vaccines During the 2024–2025 Season: Subgroup Analyses by Virus Subtype, Time Since Vaccination, and Diagnostic Method

**DOI:** 10.3390/vaccines14010102

**Published:** 2026-01-21

**Authors:** Yu Jung Choi, Jungmin Lee, Joon Young Song, Seong-Heon Wie, Jacob Lee, Jin-Soo Lee, Hye Won Jeong, Joong Sik Eom, Jang Wook Sohn, Young Kyung Yoon, Won Suk Choi, Eliel Nham, Jin Gu Yoon, Ji Yun Noh, Man-Seong Park, Hee Jin Cheong

**Affiliations:** 1 Division of Infectious Diseases, Department of Internal Medicine, Korea University College of Medicine, Seoul 02841, Republic of Korea; 2 Vaccine Innovation Center-KU Medicine (VIC-K), Seoul 02841, Republic of Korea; 3 Department of Microbiology, Institute for Viral Diseases, Korea University College of Medicine, Seoul 02841, Republic of Korea; 4 Division of Infectious Diseases, Department of Internal Medicine, St. Vincent’s Hospital, College of Medicine, The Catholic University of Korea, Suwon 16247, Republic of Korea; 5 Division of Infectious Diseases, Department of Internal Medicine, Kangnam Sacred Heart Hospital, Hallym University College of Medicine, Seoul 07441, Republic of Korea; 6 Division of Infectious Diseases, Department of Internal Medicine, Inha University School of Medicine, Incheon 22332, Republic of Korea; 7 Department of Internal Medicine, Chungbuk National University College of Medicine, Cheongju 28644, Republic of Korea; 8 Division of Infectious Diseases, Department of Internal Medicine, Gil Medical Center, Gachon University College of Medicine, Incheon 21565, Republic of Korea

**Keywords:** seasonal influenza, influenza vaccine, vaccine effectiveness, test-negative design

## Abstract

**Background/Objectives**: Despite high vaccination coverage, influenza remains a public health concern in South Korea, particularly in older adults. Continuous evaluation of vaccine effectiveness (VE) is essential to optimize immunization strategies. **Methods**: This study evaluated seasonal influenza VE for preventing laboratory-confirmed influenza using a test-negative design through a hospital-based influenza surveillance system in South Korea from 1 November 2024, to 30 April 2025. Demographic and clinical information was collected through questionnaire surveys and electronic medical records. Influenza was diagnosed using rapid antigen tests (RATs) and reverse transcription polymerase chain reaction (RT-qPCR), and vaccine effectiveness was analyzed using multivariable logistic regression. **Results**: In total, 3954 participants were included, with 1977 influenza-positive cases and 1977 test-negative controls. Influenza A and B accounted for 93.1% and 7.0% of cases, respectively. The adjusted overall VE was 20.4% (95% confidence interval [CI], 8.2–30.9; *p* = 0.002). VE was higher in adults aged 50–64 years (46.8%) than in those aged ≥65 years (18.8%). VE was 19.9% against influenza A and 45.7% against A/H3N2. VE was higher among individuals tested using RT-qPCR than among those tested using RATs (21.5% vs. 15.7%), and was also greater during the early period than during the late period (20.5% vs. 11.4%). Vaccination did not reduce influenza-associated hospitalization risk (VE, 17.3%; 95% CI, −9.3 to 37.4). A significant reduction in hospitalization risk was observed in adults aged 50–64 years (VE, 46.8%), with no significant benefit in those aged ≥65 years. **Conclusions**: The 2024–2025 seasonal influenza vaccine provided moderate protection against laboratory-confirmed influenza in adults, with higher effectiveness in those aged 50–64 years.

## 1. Introduction

Influenza, a respiratory virus that continues to pose a serious threat to public health, requires ongoing vigilance despite the significant attention given to SARS-CoV-2. During the 2024–2025 season, South Korea experienced the highest incidence of influenza-like illness (ILI) in the last 8 years, characterized by two separate epidemic peaks [1]. The predominant circulating strain was A/H1N1pdm09 in the early phase, followed by influenza B viruses in the later stages.

Vaccination against influenza remains the most effective strategy for reducing the disease burden associated with influenza. In interim analyses conducted earlier in the same influenza season in South Korea, using data from the same national surveillance system as the present study, only a modest protective effect was observed, in contrast to the higher interim estimates of approximately 34–52% reported in other countries [2,3,4]. Unlike many countries that rely almost exclusively on real-time reverse transcription polymerase chain reaction (RT-qPCR) to diagnose influenza, rapid antigen tests (RATs) are widely used in clinical practice in Korea to detect influenza because they are less expensive, more accessible, and provide faster results.

Although vaccine effectiveness (VE) is influenced by multiple factors, including host characteristics, antigenic matches, vaccination timing, and diagnostic methods, these contextual factors must be considered when interpreting and comparing VE estimates [5]. Vaccine effectiveness is particularly reduced in older adults as a consequence of immunosenescence, characterized by age-related deterioration of innate and adaptive immune cell function and impaired vaccine-induced immune responses [6,7]. In other countries, strategies such as high-dose and adjuvanted vaccines have been adopted to optimize vaccine effectiveness in this population [8].

Therefore, this study aimed to estimate the real-world VE for the 2024–2025 influenza season in South Korea using subgroup analysis according to the virus subtype, time since vaccination, and diagnostic method.

## 2. Materials and Methods

### 2.1. Data Source and Collection

The study was conducted from November 2024 to April 2025 using a hospital-based influenza surveillance network (hospital-based influenza morbidity and mortality), which consists of eight university hospitals. Patients who visited outpatient clinics or emergency departments for ILI, including hospitalized patients who underwent RAT or RT-qPCR testing, were enrolled in this study. ILI was defined as the presence of sudden fever of ≥37.8 °C along with symptoms such as cough, sore throat, or nasal discharge/congestion. Individuals younger than 18 years of age were excluded.

### 2.2. Vaccination

Vaccination history was verified using self-reported questionnaires and medical records and cross-checked with the national immunization registry maintained by the Korea Disease Control and Prevention Agency. Vaccination status was considered valid if the vaccine was administered at least 14 days before the diagnosis date. In South Korea, the recommended quadrivalent influenza vaccines are administered in accordance with World Health Organization recommendations. The egg-based quadrivalent inactivated vaccines used in South Korea included the A/Victoria/4897/2022 (H1N1)pdm09-like virus, the A/Thailand/8/2022 (H3N2)-like virus, the B/Austria/1359417/2021 (B/Victoria lineage)-like virus, and the B/Phuket/3073/2013 (B/Yamagata lineage)-like virus [9]. The cell-culture or recombinant-based vaccines licensed in South Korea included the A/Wisconsin/67/2022 (H1N1)pdm09-like virus, the A/Massachusetts/18/2022 (H3N2)-like virus, the B/Austria/1359417/2021 (B/Victoria lineage)-like virus, and the B/Phuket/3073/2013 (B/Yamagata lineage)-like virus. In South Korea, the MF59-adjuvanted quadrivalent influenza vaccine (Fluad^®^ Quad) has been introduced and used since 2023, followed by the introduction of the high-dose influenza vaccine (Efluelda^®^) in 2024; both vaccines contain the same WHO-recommended strains as standard egg-based influenza vaccines.

### 2.3. Diagnostic Tools

Influenza was identified using either RAT or RT-qPCR according to standard procedures, with samples collected within 7 days of symptom onset. Despite the relatively low sensitivity of RATs, they were included in this study to reflect real-world clinical practice in Korea, where RATs are widely used. To address the issue of reduced sensitivity, only patients who developed symptoms within 48 h were included to minimize false-negative results.

### 2.4. Statistical Analysis

A test-negative case–control design was used to evaluate VE. Each influenza-positive individual was matched 1:1 with a corresponding influenza-negative control based on age, sex, and date of diagnosis. VE was derived using the formula: VE = (1 − odds ratio) × 100%. Logistic regression was used to estimate the odds ratio, adjusting for potential confounders, including age, sex, and underlying health conditions, to control for differences between groups. Vaccine effectiveness against hospitalization was assessed using a test-negative design restricted to hospitalized patients presenting with ILI. To minimize misclassification and avoid overestimating hospitalization VE, we excluded hospital-onset influenza cases, defined as those diagnosed ≥72 h after admission. Statistical significance was set at *p* < 0.05. All analyses were performed using SPSS version 20.0 (IBM Corp., Armonk, NY, USA).

### 2.5. Ethics Statement

This study was approved by the Institutional Review Board of each participating hospital, as detailed in the Institutional Review Board Statement. All participants provided written informed consent before enrollment in the study.

## 3. Results

In total, 3954 participants were included from November 2024 to April 2025, comprising 1977 influenza-positive cases and 1977 test-negative controls (Supplementary Figure S1). The overall results of influenza testing using RAT and RT-qPCR are shown in Table 1. Among the influenza-positive cases, 1840 (93.1%) were influenza A and 139 (7.0%) were influenza B. Both types, A and B, were detected using the RAT in two cases. Among the influenza A cases with available subtype data, 135 (70.0%) were classified as A/H1N1 and 58 (30.1%) as A/H3N2.

### 3.1. Baseline Characteristics Between Groups

The demographic and clinical characteristics of the two groups are shown in Table 2. No significant differences were noted between the groups in terms of the month of enrollment, age, or sex. Most participants were diagnosed in December and January, accounting for 24.7% and 66.1% of cases, respectively. Individuals aged ≥65 years comprised 42.4% of the study population. The test-positive group had a significantly higher proportion of patients without underlying conditions, which may have been due to more active testing among individuals with comorbidities. The prevalence of solid malignancies was significantly higher in the test-negative group (10.0% vs. 14.7%). No significant differences were observed in the clinical outcomes between the two groups, except for hospital admission. Patients with negative test results tended to be hospitalized more frequently because of uncertainty regarding their clinical courses.

### 3.2. Vaccine Status of Study Participants

In total, 47.6% (1883) had received an influenza vaccination, and the vaccine type was identified for all individuals except one. A standard quadrivalent egg-based influenza vaccine was administered to 64.1% (1206) of the participants, and 35.9% (676) were vaccinated with the cell-based quadrivalent influenza vaccine. None of the study participants were vaccinated with an adjuvanted or high-dose influenza vaccine. Vaccination coverage was lower in the test-positive group than in the test-negative group (45.8% vs. 49.4%).

### 3.3. Vaccine Effectiveness by Age Group and Subtype

Detailed estimates of VE stratified by age group and virus subtype are shown in Table 3. The adjusted overall VE was 20.4% (95% confidence interval [CI], 8.2 to 30.9; *p* = 0.002). In the age-stratified analysis, a significantly higher VE of 46.8% was observed in the 50–64 age group (*p* < 0.001), whereas VE in those aged ≥65 years was lower at 18.8%.

According to virus subtype, the overall VE was 19.9% against influenza A (95% CI, 7.4–30.7; *p* = 0.003) and 18.4% against A/H1N1, which was not statistically significant. In contrast, VE against A/H3N2 was significantly higher at 45.7% (95% CI, 2.3–69.8; *p* = 0.042), despite the small sample size. The lower overall VE against influenza A reflects the predominance of A/H1N1 cases (*n* = 135), which were associated with lower vaccine effectiveness, compared with A/H3N2 cases, for which fewer cases were observed (*n* = 58). For influenza B, a VE of 51.3% was observed; however, this result was not statistically significant.

### 3.4. Vaccine Effectiveness for Hospitalization Prevention

Overall, influenza vaccination did not confer a statistically significant reduction in influenza-associated hospitalizations (Table 4). After excluding 403 hospital-onset cases from 1591 hospitalized patients, the hospitalization risk was modestly lower among vaccinated individuals, corresponding to a non-significant VE of 17.3%. Age-stratified analyses revealed that only adults aged 50–64 years exhibited a marginal and relatively higher reduction in hospitalization risk, with an estimated VE of 48.3% (95% CI, −0.2 to 73.3; *p* = 0.051). Although this point estimate suggests potentially meaningful protection in this age group, the wide confidence interval indicates borderline statistical significance. In contrast, adults aged ≥65 years showed no significant vaccine effectiveness without measurable reduction in hospitalization risk among older adults.

### 3.5. Real-World Vaccine Effectiveness, Stratified by Time Since Vaccination and Diagnostic Method

VE declined as the time since vaccination increased. VE was 20.5% (95% CI, 7.8–31.4; *p* = 0.002) in the early period (Nov–Jan) group but decreased to −11.4% in the late period (Feb–Apr) group, which was not statistically significant (Table 5).

RT-qPCR was performed in 1272 cases (32.2%), whereas RATs were conducted in 2997 cases (75.8%). VE estimated among patients tested with RATs was 15.7 (95% CI, 0.9–31.7; *p* = 0.039), which was lower than the VE of 21.5 (95% CI, −22.2–39.8; *p* = 0.072) observed among patients tested with RT-qPCR.

In subgroup analysis stratified by vaccine platform, VE against laboratory-confirmed influenza was estimated at 31.1% (95% CI, 18.0–42.2; *p* = <0.001) for the egg-based vaccines and 3.8% (95% CI, −15.3–198.8; *p* = 0.672) for the cell-based vaccines, with no statistically significant difference observed between the two platforms.

### 3.6. Laboratory Surveillance of Influenza Viruses and Matching Status with Vaccine Strains

**Figure 1:**
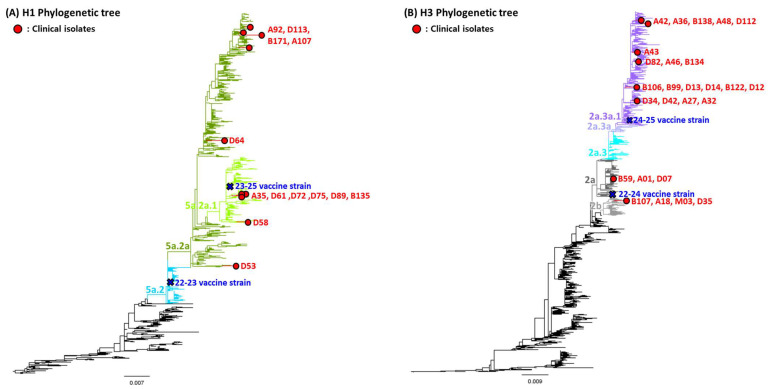
Phylogenetic analysis of the influenza A virus isolates. The phylogenetic tree was constructed using Nextclade version 3.15.3. Isolates were marked with red dots.

Forty influenza viruses were genetically characterized, comprising 13 A/H1N1, 26 A/H3N2, and one influenza B virus. Among the A/H1N1 viruses, approximately half (7/13, 54%) belonged to HA clade 5a.2a, whereas the remaining six (46%) were classified within HA clade 5a.2a.1, which matched the vaccine strain (Figure 1). For the A/H3N2 viruses, most (73%) belonged to HA clade 2a.3a.1, which is the same clade as the A/H3N2 component of the 2024–2025 influenza vaccine. One influenza B virus was identified as clade V1A.3a.2, matching the Victoria lineage of the vaccine strain.

## 4. Discussion

This study assessed the real-world effectiveness of the seasonal influenza vaccine in Korean adults during the 2024–2025 season. Overall, the influenza vaccine showed a preventive effect of 20.4% (95% CI, 8.2 to 30.9; *p* = 0.002) and a higher rate of 46.8% in the 50–64 age group. When stratified by virus subtype, VE was 19.7% against influenza A overall and substantially higher at 45.7% against A/H3N2; the lower overall effectiveness primarily reflected the predominance of A/H1N1 cases, which were associated with relatively lower VE. In contrast, vaccination did not significantly reduce influenza-associated hospitalization risk overall; only adults aged 50–64 years showed a marginal reduction, with no measurable benefit in those aged ≥65 years.

Influenza VE decreases with age, and the findings of lower VE among individuals aged ≥65 years were consistent with the analyses reported by the Centers for Disease Control and Prevention (CDC) and Eurosurveillance during the same season [2,3,10]. In Korea, unlike standard-dose vaccines, high-dose or MF59-adjuvanted influenza vaccines are not provided free of charge, which may lead to a low VE among older adults. In this study, subgroup analysis by vaccine type could not be conducted because none of the participants had received a highly immunogenic vaccine. However, the US CDC recommends highly immunogenic formulations, such as high-dose, recombinant, or adjuvanted influenza vaccines, as the preferred options for individuals aged ≥65 years to enhance vaccine efficacy in this population [7]. This policy emphasizes the importance of selecting vaccines with enhanced immunogenicity, especially in the older population, who are at a higher risk of severe influenza-related complications.

In the 2024–2025 season, the overall VE estimated in this study was lower than the interim VE estimates reported by the US CDC (32–60%) and the contemporaneous estimates reported by Eurosurveillance (34–52%) [2,3]. This study investigated VE using RAT and RT-PCR. Although the RAT has excellent specificity, its sensitivity is relatively low and has been reported to be 69% in a meta-analysis [11]. This lower sensitivity may have led to the underdetection of true influenza cases, thereby underestimating the VE [12,13]. Despite these limitations, it is important to include diagnostic methods when estimating VE in South Korea. In real-world practice, the RAT is commonly performed first, and a positive result is sufficient for clinical diagnosis and treatment initiation. RAT was used in 75.8% of the tested cases, whereas RT-PCR was performed in only 24.2% of the cases. Although the influenza VE estimated using RAT was somewhat lower than that assessed using PCR-based methods, the difference was not as pronounced as expected. Importantly, this finding should not be interpreted as indicating true biological differences in vaccine-induced protection by test type but rather as reflecting differences in diagnostic sensitivity across testing methods.

Although many previous studies have demonstrated that influenza vaccination significantly reduces severe outcomes [14,15,16], our findings did not show a statistically significant reduction in influenza-associated hospitalizations. In this study, vaccination was associated with a non-significant 17.3% decrease in hospitalization risk. This lack of significance may partly reflect the high proportion of severe, hospitalization-requiring influenza cases among adults aged ≥65 years, an age group in which the vaccine showed no measurable protective effect. These observations underscore an important limitation of current vaccine platforms, particularly their modest protection against infection and suboptimal performance in older adults with immunosenescence. Given that secretory immunoglobulin A plays a central role in cross-protective mucosal immunity, mucosal vaccine development has gained momentum to overcome the constraints of parenterally administered inactivated vaccines, which offer limited heterologous protection [17]. These findings highlight the need for next-generation vaccine platforms designed to elicit robust mucosal and broad cross-protective immune responses. Furthermore, detailed analyses comparing standard-dose, high-dose, and adjuvanted formulations are warranted to refine national immunization strategies and guide evidence-based policy decisions.

The estimated VE for H3N2 and influenza B was relatively high in the older subgroup at 41.8% and 73.4%, respectively, although the differences were not statistically significant. This suggests that multiple factors, including age, underlying health conditions, and matching status between the vaccine and circulating strains, may have influenced the observed VE in this group. A study analyzing 10 influenza seasons in Korea showed that VE differed according to matching status [18]. The US CDC and Eurosurveillance reports showed a close genetic alignment between circulating viruses and vaccine components during the 2024–2025 season. The A(H3N2) virus was predominantly from HA clade 2a.3a.1 (US CDC: all 286 sequence-confirmed isolates in 2a.3a.1; ECDC: J.2 subclade within 2a.3a.1). The A(H1N1)pdm09 virus largely clustered in clade 5a.2a, with a smaller proportion of 5a.2a.1 (US CDC: mixed 5a.2a and 5a.2a.1 among 158 sequences; ECDC: >80% C.1.9 within 5a.2a) [3]. This study demonstrated a higher proportion of vaccine-matched strains among H3N2 viruses than among H1N1 viruses in South Korea. This finding may partly explain the greater effectiveness of the vaccine against the H3N2 subtype. For influenza B, all virus isolates belonged to the B/Victoria lineage (V1A.3a.2 subclade), which remained antigenically well-matched to the vaccine strain, and no B/Yamagata lineage viruses were detected according to Eurosurveillance reports [4]. Within the H3N2 clade 2a.3a.1, sequence diversity was primarily concentrated along lineage-defining branches (e.g., J.2), accompanied by sporadic amino acid substitutions in the HA antigenic regions that may modestly diminish antigenic recognition within certain viral subsets. In contrast, the B/Victoria lineage (V1A.3a.2) remained antigenically stable, which may account for the comparatively higher VE observed for influenza B and the greater variability in VE against H3N2. The global predominance of the B/Victoria lineage, with no B/Yamagata detection, supports the high influenza B VE (73–88%) reported by Eurosurveillance and reinforces the rationale for transitioning from quadrivalent to trivalent vaccine formulations.

The major strength of this study is that the influenza VE was evaluated through well-established hospital-based networks, which enabled real-time assessment. However, this study had certain limitations. First, only a limited number of viral isolates were available for laboratory surveillance analysis, which constrained a more robust assessment of vaccine-circulating strain matching. Second, the sample size for certain subgroups, such as individuals vaccinated with highly immunogenic vaccines, was too small to allow meaningful subgroup analysis. In addition, although differences in vaccine effectiveness estimates by vaccine platform were observed, these differences did not reach statistical significance, likely owing to limited sample sizes. Moreover, the cell-culture influenza vaccines used in South Korea are not entirely egg-free, as they rely on egg-derived seed viruses. Furthermore, reductions in vaccine effectiveness attributable to egg-adapted mutations predominantly affect A/H3N2 strains; however, during the study season, A/H1N1 was the dominant circulating strain, which may have limited our ability to detect meaningful platform-specific differences. Consequently, potential antigenic differences between egg-based and cell-based vaccines may be attenuated, and vaccine platform–specific comparisons should therefore be interpreted with caution [19,20]. Third, the reliance on RAT in some cases may have led to misclassification of influenza infection status due to its lower sensitivity compared to PCR, potentially underestimating VE. Fourth, the inclusion of only university hospitals may have led to an overrepresentation of more severe cases, thereby limiting the generalizability of the findings. Finally, the observational nature of this study inherently limits the ability to establish causality, and the possibility that residual confounding factors may have influenced the results cannot be completely ruled out. Although vaccination history in previous seasons may influence vaccine effectiveness, we were unable to assess its impact in this study. Future studies should include laboratory surveillance data and adopt a more systematic and in-depth approach to strengthen these findings.

## 5. Conclusions

In conclusion, these findings indicate that the 2024–2025 seasonal influenza vaccine provided moderate protection among adults but showed reduced effectiveness in older adults (≥65 years) and during the latter part of the season. These results underscore the need to optimize vaccination strategies by considering seasonal dynamics and age-related declines in vaccine-induced protection.

## Figures and Tables

**Table 1:** Comparison of influenza RAT and RT-qPCR results.

		RT-qPCR						
		Negative	A/H1N1	A/H3N2	A (Non-Typed)	B	Not Performed	Total
RAT	Negative	**160**	41	11	16	2	**1403**	1633
	Type A	10	41	12	19	0	1176	1258
	Type B	2	0	0	0	1	101	104
	Type A and B	0	0	0	0	0	2	2
	Not Performed	**414**	53	35	424	31	0	957
	Total	586	135	58	459	34	2682	3954

Bold cases were defined as influenza-negative.

**Table 2:** Baseline characteristics of study participants.

	No. of Participants (*n* = 3954)	Test-Positive Cases (*n* = 1977)	Test-Negative Controls (*n* = 1977)	*p*-Value
**Month of enrollment**				1.000
November 2024	20 (0.5)	10 (0.5)	10 (0.5)	
December 2024	978 (24.7)	489 (24.7)	489 (24.7)	
January 2025	2612 (66.1)	1306 (66.1)	1306 (66.1)	
February 2025	172 (4.4)	86 (4.4)	86 (4.4)	
March 2025	78 (2.0)	39 (2.0)	39 (2.0)	
April 2025	94 (2.4)	47 (2.4)	47 (2.4)	
**Age group**				1.000
19–49 yrs	1488 (37.6)	744 (37.6)	744 (37.6)	
50–64 yrs	788 (19.9)	397 (19.9)	397 (19.9)	
≥65 yrs	1678 (42.4)	839 (42.4)	839 (42.4)	
**Sex**				0.655
Female	2118 (53.6)	1052 (53.2)	1066 (53.9)	
Male	1836 (46.4)	925 (46.8)	911 (46.1)	
**Comorbidities**				
None	1775 (44.9)	935 (47.3)	840 (42.5)	0.002 *
Diabetes mellitus	864 (21.9)	441 (22.3)	423 (21.4)	0.488
Cardiovascular disease	482 (12.2)	243 (12.3)	239 (12.1)	0.846
Chronic pulmonary disease	403 (10.2)	206 (10.4)	197 (10.0)	0.636
Chronic renal disease	294 (7.4)	155 (7.8)	139 (7.0)	0.332
Chronic liver disease	143 (3.6)	68 (3.4)	75 (3.8)	0.551
Chronic neurological disease	503 (12.7)	237 (12.0)	266 (13.5)	0.164
Solid malignancy	488 (12.3)	197 (10.0)	291 (14.7)	<0.001 *
Hematologic malignancy	132 (3.3)	59 (3.0)	73 (3.7)	0.215
Immunosuppressive agent use	93 (2.4)	43 (2.2)	50 (2.5)	0.463
AIDS	3 (0.1)	2 (0.1)	1 (0.1)	0.564
Autoimmune disease	83 (2.1)	42 (2.1)	41 (2.1)	0.912
**Influenza vaccination**	1883 (47.6)	906 (45.8)	977 (49.4)	0.024 *
**Clinical outcomes**				
Admission	1591 (40.2)	687 (34.7)	904 (45.7)	<0.001 *
ICU Admission	336 (8.5)	155 (7.8)	181 (9.2)	0.203
Mechanical ventilation	158 (4.0)	76 (3.8)	82 (4.1)	0.539
Mortality	105 (2.7)	47 (2.4)	58 (2.9)	0.467

Abbreviations: ICU, intensive care unit; AIDS, acquired immunodeficiency syndrome. * *p* < 0.05.

**Table 3:** Estimated influenza vaccine effectiveness by subtype and age group.

	Test-Positive, Vaccinated/Total (%)	Test-Negative, Vaccinated/Total (%)	Adjusted VE (95% CI) (%)	*p*-Value
**Influenza**				
Overall	906/1977 (45.8)	977/1977 (49.4)	20·4 (8.2 to 30.9)	0.002 *
19–49 yrs	223/744 (30.0)	226/744 (30.4)	5·9 (−18.4 to 25.2)	0.604
50–64 yrs	82/394 (20.8)	123/394 (31.2)	46·8 (25.6 to 62.0)	<0.001 *
≥65 yrs	601/839 (71.6)	628/839 (74.9)	18·8 (−1.1 to 34.9)	0.063
**Influenza A**				
Overall	863/1840 (46.9)	977/1977 (49.4)	19·7 (7.2 to 30.5)	0.003 *
19–49 yrs	199/657 (30.3)	226/744 (30.4)	5·7 (−19.5 to 25.6)	0.628
50–64 yrs	77/366 (21.0)	123/394 (31.2)	46·6 (24.8 to 62.0)	<0.001 *
≥65 yrs	587/817 (71.8)	628/839 (74.9)	17·9 (−2.5 to 34.2)	0.082
**Influenza A/H1N1**				
Overall	79/135 (58.5)	977/1977 (49.4)	18·4 (−22.6 to 45.7)	0.327
19–49 yrs	1/12 (8.3)	226/744 (30.4)	79·1 (−66.1 to 97.4)	0.139
50–64 yrs	4/24 (16.7)	123/394 (31.2)	59·1 (−30.5 to 87.2)	0.131
≥65 yrs	74/99 (74.7)	628/839 (74.9)	−4·9 (−72.5 to 36.2)	0.850
**Influenza A/H3N2**				
Overall	28/58 (48.3)	977/1977 (49.4)	45·7 (2.3 to 69.8)	0.042 *
19–49 yrs	0/5 (0.0)	226/744 (30.4)	N/A	N/A
50–64 yrs	3/13 (23.1)	123/394 (31.2)	29·9 (−175.3 to 82.2)	0.611
≥65 yrs	25/40 (62.5)	628/839 (74.9)	41·8 (−14.5 to 70.4)	0.117
**Influenza B**				
Overall	45/139 (32.4)	977/1977 (49.4)	51·3 (−12.2 to 78.9)	0.091
19–49 yrs	25/88 (28.4)	226/744 (30.4)	34·5 (−86.2 to 76.9)	0.428
50–64 yrs	5/28 (17.9)	123/394 (31.2)	67·8 (−2.536 to 97.1)	0.354
≥65 yrs	15/23 (65.2)	628/839 (74.9)	73·4 (−37.6 to 94.8)	0.114

Abbreviations: VE: vaccine effectiveness. * *p* < 0.05.

**Table 4:** Estimated effectiveness of the influenza vaccine against influenza-associated hospitalization.

	Case, Vaccinated/Total (%)	Control, Vaccinated/Total (%)	Adjusted VE (95% CI) (%)	*p*-Value
Overall	265/492 † (53.9)	365/696 † (52.4)	17·3 (−9.3 to 37.4)	0.181
19–49 yrs	12/64 (18.8)	26/136 (19.1)	7·1 (−121.5 to 89.4)	0.861
50–64 yrs	18/108 (16.7)	38/154 (24.7)	48·3 (−0.2 to 73.3)	0.051
≥65 yrs	235/320 (73.4)	301/406 (74.1)	10·1 (−26.6 to 36.1)	0.543

Abbreviations: VE, vaccine effectiveness. † Of the 1591 hospitalized patients, 403 nosocomial influenza cases were excluded.

**Table 5:** Overall estimated influenza vaccine effectiveness stratified by time since vaccination and diagnostic method.

		Test-Positive, Vaccinated/Total (%)	Test-Negative, Vaccinated/Total (%)	Adjusted VE (95% CI) (%)	*p*-Value
Time since vaccination	Early period (Nov–Jan)	833/1805 (46.1)	898/1805 (49.8)	20.5 (7.8 to 31.4)	0.002 *
	Late period (Feb–Apr)	37/86 (43.0)	34/86 (37.5)	−11.4 (−136.2 to 47.4)	0.778
Diagnostic method	RAT	641/1434 (44.7)	750/1563 (48.0)	15.7 (0.9 to 28.4)	0.039 *
	RT-qPCR	346/698 (49.6)	300/574 (52.3)	21.5 (−2.2 to 39.8)	0.072

Abbreviations: VE, vaccine effectiveness; RAT, rapid antigen test; RT-qPCR, reverse transcription polymerase chain reaction. * *p* < 0.05.

## Data Availability

The data from this study are not accessible to the public.

## Supplementary Materials

The following supporting information can be downloaded at: https://www.mdpi.com/article/10.3390/vaccines14010102/s1, Figure S1: Flowchart of study participants.

## Abbreviations

The following abbreviations are used in this manuscript:

VE	vaccine effectiveness
CDC	Centers for Disease Control and Prevention
