# Supplementary figures and images for: Real-World Effectiveness of Seasonal Influenza Vaccines During the 2024–2025 Season: Subgroup Analyses by Virus Subtype, Time Since Vaccination, and Diagnostic Method
Source: Vaccines (Basel). 2026 Jan 21;14(1):102. doi: 10.3390/vaccines14010102 (PMC12846531; doi:10.3390/vaccines14010102)

Supplementary Figure S1

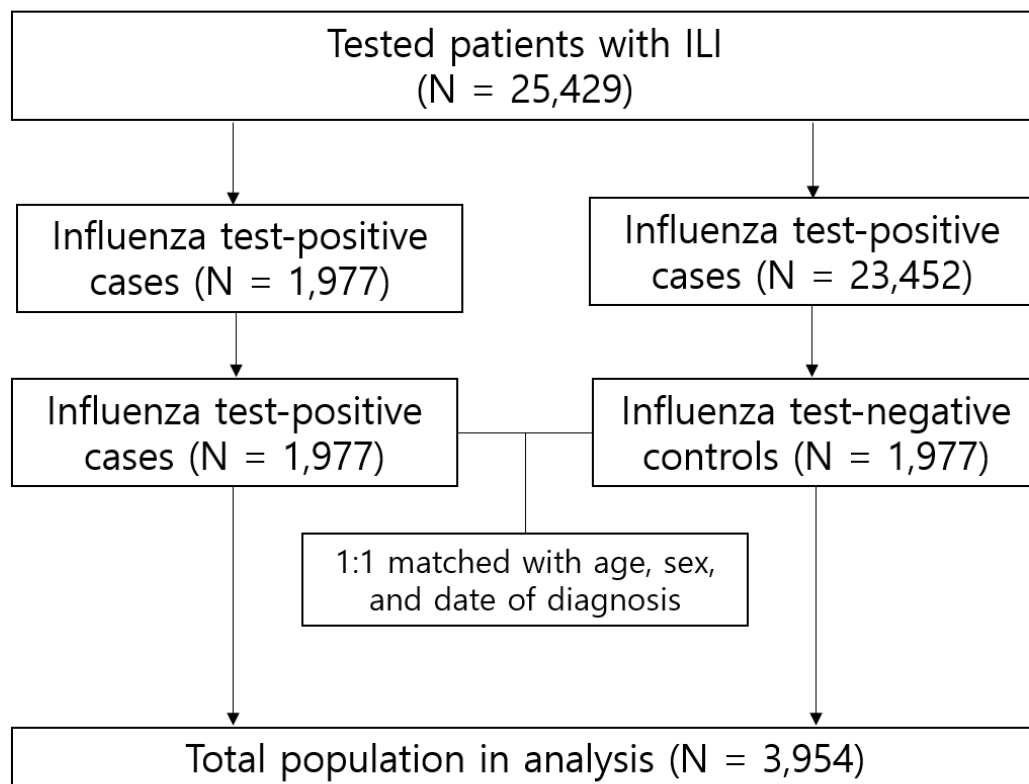

Supplement: Supplementary file 1 [file vaccines-14-00102-s001.zip › vaccines-4080037-Supplementary.pdf]
